# Reference values and the effect of clinical parameters on thyroid hormone levels during early pregnancy

**DOI:** 10.1042/BSR20202296

**Published:** 2021-01-06

**Authors:** Huijia Lin, Mudan Ye, Zhenwen Zhou, Lianxiong Yuan, Gendie E. Lash, Guanglan Zhang, Li Li

**Affiliations:** 1 Department of Obstetrics and Gynecology, Guangzhou Women and Children’s Medical Center, Guangzhou Medical University, Guangzhou, China; 2 Department of Laboratory Medicine, Guangzhou Women and Children’s Medical Center, Guangzhou Medical University, Guangzhou, China; 3 Department of Biostatistics, Sun Yat-sen University, Guangzhou, China; 4 Guangzhou Institute of Pediatrics, Guangzhou Women and Children’s Medical Center, Guangzhou Medical University, Guangzhou, China

**Keywords:** Body Mass Index, Human Chorionic Gonadotropin, Lipid, Pregnancy, Thyroid

## Abstract

Objective: Thyroid dysfunction is a common endocrine problem during pregnancy; correct diagnosis and appropriate treatments are essential to avoid adverse pregnancy outcomes. Besides, it is vital to identify and quantify the major risk factors for gestational thyroid dysfunction, including thyroid autoimmunity, human chorionic gonadotropin (HCG) concentration, body mass index (BMI) and parity. The study objective was to establish reference ranges during early pregnancy and to explore the relationship between risk factors and thyroid stimulating hormone (TSH), free thyroxine (FT4) and free triiodothyroxine (FT3).

Design, patients and measurements: To establish the reference ranges of thyroid hormone during early pregnancy in China and to identify the risk factors for thyroid dysfunction, woman in the first trimester of pregnancy (4–12 weeks gestation) were recruited***.*** After excluding thyroid peroxidase antibody (TPO-Ab) positive and/or thyroglobulin antibody (TG-Ab) positive women, previous thyroid disease, a lack of iodine intake, reference values were calculated by 2.5th to 97.5th percentiles.

Results: After exclusion of TPO-Ab and/or TG-Ab positive women, reference values were as follows: TSH, 0.11–3.67 mIU/l; FT3, 3.19–5.91 pmol/l; FT4 10.95–16.79 pmol/l. Higher BMI was associated with lower FT4 concentrations (*P*=0.005). In multiple regression analysis, TSH was significantly and positively associated with TG (*P*=0.03). Maternal parity and maternal age may be risk factors for the abnormal thyroidal response to hCG concentrations.

Conclusions***:*** Our study defined first trimester-specific reference ranges for serum TSH, FT4, FT3 in a Chinese population, and demonstrated that BMI ≥23kg/m^2^, maternal parity ≥3 and maternal age ≥30 years may increase the risk of thyroid dysfunction.

## Introduction

Thyroid dysfunction is a common endocrine disease during pregnancy, approximately 0.2–0.6% of pregnant women suffer from hypothyroidism, and subclinical hypothyroidism occurs in an additional 3.5–18.0% of pregnant women [1]. The prevalence of hyperthyroidism during pregnancy is approximately 1%, of which clinical hyperthyroidism accounts for 0.4% and subclinical hyperthyroidism accounts for 0.6%, the major contributing factors are Graves disease (85%) and transient thyrotoxicosis (10%) [2,3]. For women during pregnancy, thyroid hormone deficiency can lead to adverse pregnancy outcomes, including premature delivery, placental abruption, miscarriage and gestational hypertension [4,5]. At the same time, thyroid hormone deficiency during pregnancy, especially in the first trimester, may cause adverse fetal outcomes, including low birth weight, low fetal intelligence, dysplasia of the nervous system and even stillbirth [6–10].

The release of thyroid hormone during pregnancy is affected by many factors, such as race, iodine status, urinary iodine status, obesity, age, HCG concentrations, parity and fetal sex. Due to the adverse relationship between thyroid dysfunction and adverse offspring outcomes, treatment with levothyroxine sodium (LT-4) and antithyroid drugs is essential. However, overtreatment of thyroid dysfunction, including subclinical hypothyroidism and subclinical hyperthyroidism, may also increase the risk of adverse outcomes in offspring. Therefore, the definition of hypothyroidism (OH) and hyperthyroidism is crucial. Indeed, in 2017 ATA revised the guidelines to suggest ‘Population-based trimester-specific reference ranges for serum TSH should be defined through assessment of local population data representative of a health care provider’s practice. Reference range determinations should only include pregnant women with no known thyroid disease, optimal iodine intake, and negative TPO-Ab status’. [11]. For pregnant women in the United States and parts of Europe, the upper limit of TSH reference for the first trimester of pregnancy is recommended to be 2.5 mU/l, and the upper limit of TSH reference for the second trimester and third trimester of pregnancy is 3.0 mU/l, while recent evidence suggests that in Asia, India and the Netherlands the upper reference limit should be lowered [12–18]. According to the 2017 guidelines, overt maternal hypothyroidism is defined as the presence of elevated TSH and decreased serum FT4 concentrations during gestation, or a TSH concentration exceeding 10 mIU/l regardless of FT4 concentration, and subclinical hypothyroidism is defined as the presence of elevated TSH with a normal serum FT4 concentration. At the same time, the guidelines published by ATA in 2017 do not recommend universal thyroid function screening, except for the following patients: thyroid antibody positive, >30 years of age, with a history of adverse pregnancy outcomes, multiple prior pregnancies (≥2), or morbidly obese (BMI ≥40 kg/m^2^) were recommended to be tested for serum TSH levels. The purpose of the present study was to establish the specific reference value of thyroid hormone in the first trimester of pregnancy in Chinese women, and to explore the effects of HCG, blood lipid status, BMI, age and parity on thyroid hormone status.

## Materials and methods

### Participant characteristics

About 232 women during the first trimester of pregnancy (4–12 weeks gestational age) were recruited at the Guangzhou Women and Children’s Medical Center, Guangzhou Medical University. Women were excluded if they had pre-existing thyroid disease, a history of thyroid surgery or radioactive iodine treatment, family history of thyroid dysfunction, recently taken thyroid drugs or medication which affected thyroid function such as estrogen, antidepressants and anticonvulsants, multiple gestation or assisted reproductive technology. In total 167 pregnant woman were involved in the study, all participants were invited to answer a questionnaire that included personal or family history of thyroid disease, diet, previous pregnancies (spontaneous miscarriage history, history of fetal growth restriction, previous gestational diabetes or a history of gestational hypertension, growth and development of offspring). The study was approved by the ethics committee of Guangzhou Women and Children’s Medical Center. All pregnant women signed informed consent forms.

### Sample analysis

Venous blood samples (3 ml volume) were collected into inert separating gelatinizing tubes, serum collected and stored at −20°C until required for analysis. TSH, FT4, FT3, TPO-Ab, TG-Ab, triglyceride (TG), total cholesterol (TC), low density lipoprotein (LDL), high density lipoprotein (HDL) and HCG levels were measured in the blood serum of all subjects. TSH, FT4, FT3, TPO-Ab, TG-Ab and HCG levels were measured in all subjects using electrochemiluminescence immunoassay in an Abbott I2000 analyzer. The functional sensitivity of TSH is <0.002 μIU/ml, the laboratory reference range of TSH is 0.35–4.94 mIU/l, and the laboratory reference range of FT4 and FT3 is 9.01–19.05 pmol/l and 2.63–5.70 pmol/l, respectively. The laboratory reference range of TG-Ab positive is >4.11 IU/ml, and the laboratory reference range of TPO-Ab positive is >5.61 IU/ml. The level of HCG varies dependent on gestational age, and the laboratory reference range of 1–10 weeks is 202–231000 mIU/ml. TG, TC, LDL and HDL in all subjects were measured using a HITACHI 7600-200 analyzer. The laboratory reference range for TG, TC and HDL were 0.23–1.70 mmol/l, 3.4–5.2 mmol/l and 0.88–1.80 mmol/l, respectively, and the laboratory reference range for LDL is <3.37 mmol/l. Parity was assessed as the number of pregnancies, not including losses. All participants were not wearing shoes or heavy clothing for weight and height measurements, and BMI (kg/m^2^) was calculated-a measure of body fat based on height and weight. According to the World Health Organization (WHO) appropriate body-mass index for Asian populations, the maternal BMI was divided into three categories: underweight (<18.5 kg/m^2^), normal weight (18.5–22.9 kg/m^2^), overweight (≥23 kg/m^2^) [19].

### Statistical analyses

For TSH, FT4 and FT3 analyses, the 2.5th and 97.5th percentiles were defined for all reference women excluding TPO-Ab and/or TG-Ab positive women. The association between HCG and thyroid hormone concentrations was studied using linear regression models, and the risk factors, including maternal age, BMI and parity were studied using multivariable logistic regression models. *P*<0.05 was considered significant in all statistical tests. All statistical analyses were stored in a Microsoft Excel database and performed with SPSS version 17.0 software.

## Results

**Figure 1:**
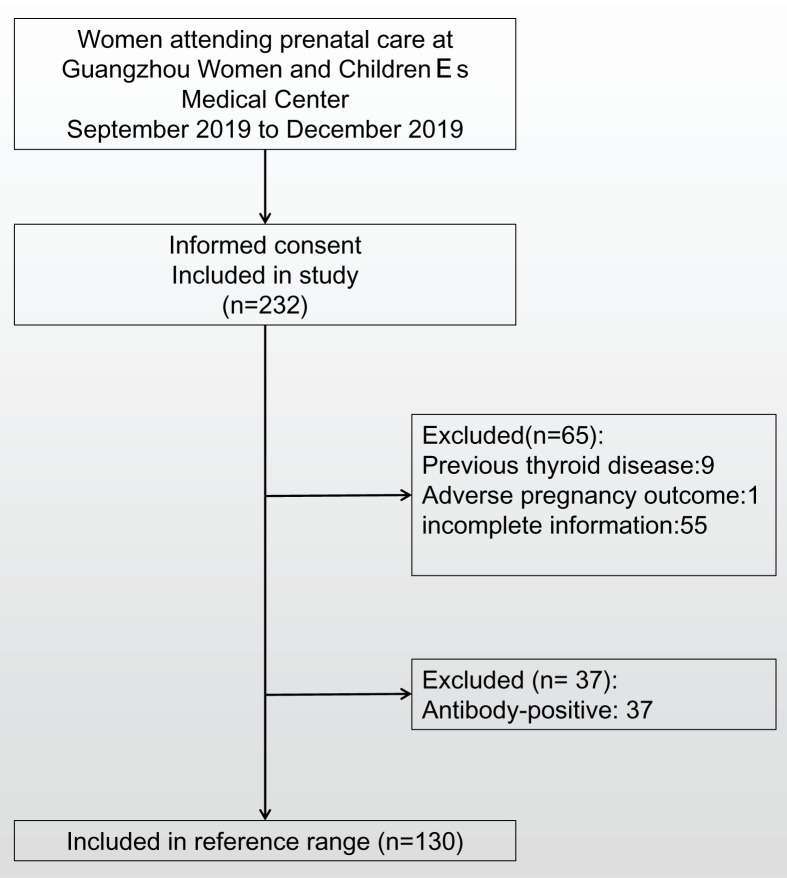
Study flow diagram Selection process of study.

From September 2019 to December 2019, 232 women in the first trimester of pregnancy were enrolled in the study. We excluded 9 women who had a history of thyroid dysfunction, 1 woman who had a miscarriage, and 55 women who did not have complete information. To establish the first trimester reference range in a Chinese cohort and compared with the 2017 ATA recommended reference range we excluded samples with positive TPO-Ab and TG-Ab status. On this basis 130 samples were included in the final analysis (Figure 1) and the basic characteristics of the 130 participants are shown in Table 1. We calculated the multiple of lower (2.5th percentile) and upper (97.5th percentile) limits for thyroid hormones. The reference range values are described in Table 2 along the 95% CI. The reference range of TSH in the first trimester was 0.11–4.06 mIU/l, lower than the upper limit of the laboratory, and a significant decrease in TSH was observed after 7 weeks of gestation. In addition, the FT4 and FT3 reference ranges during early pregnancy were 10.95–16.79 pmol/l and 3.19–5.91 pmol/L, respectively.

**Table 1:** Clinical characteristics of the study population (*n*=130)

Subject	Value
Age (years)	30.10 (22.30–40.00)
Weight (kg)	54.36 (41.00–80.00)
Height (m)	1.61 (1.50–1.71)
BMI (kg/m^2^)	21.03 (16.41–29.38)
≤18.5 kg/m^2^ (number (%))	24 (18.46%)
18.5–23 kg/m^2^ (number (%))	78 (60.00%)
≥23 kg/m^2^ (number (%))	28 (21.54%)
Gestational age (weeks)	6.89 (4.37–11.2)
HCG levels (mIU/ml)	52702.71 (754.24–216576.39)
Diet preference	
Spicy (number (%))	30(23.08%)
Light (number (%))	78(60.00%)
No preference (number (%))	22(16.92%)
Parity (number of pregnancies, not including losses)	
1 (number (%))	47 (36.15%)
2 (number (%))	70 (53.85%)
≥3 (number (%))	13 (10.00%)
Lipid	
Triglyceride (TG: mmol/l)	1.07 (0.52–2.46)
Total cholesterol (TC: mmol/l)	4.28 (3.03–6.00)
high-density lipoprotein (HDL-C: mmol/l)	1.40 (0.96–2.10)
Low-density lipoprotein (LDL-C: mmol/l)	2.34(1.45–3.70)

**Table 2:** First trimester-specific reference ranges of thyroid function in pregnant Chinese women without TPO-Ab and/or TG-ab positive (*n*=130)

Group	No.	TSH (mIU/l)	FT3 (pmol/l)	FT4 (pmol/l)
Laboratory		0.35–4.94	2.63–5.70	9.01–19.05
Total	130	0.11–3.67	3.19–5.91	10.95–16.79
Gestation 4–6 weeks	62	0.12–4.66	3.70–7.62	10.98–17.04
Gestation 7–12 weeks	68	0.06–3.74	3.79–5.33	10.56–17.39

**Figure 2:**
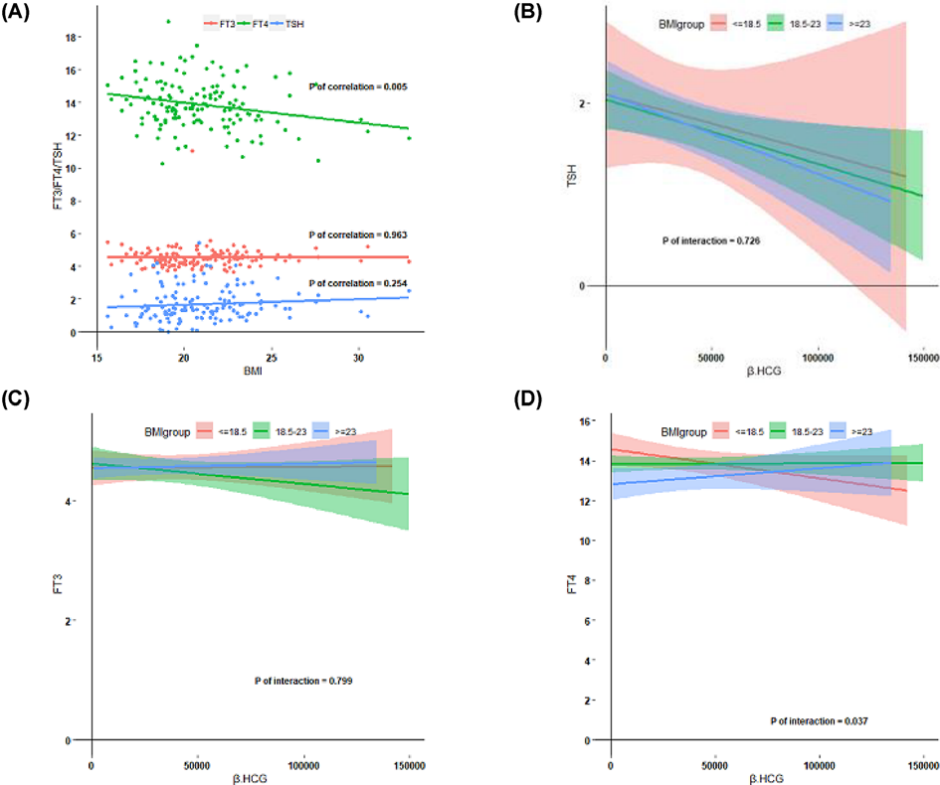
Association between thyroid hormone and BMI Data are shown as the 95% confidence intervals for TSH, FT4, FT3 according to BMI category (**A**) and HCG mediated thyroid stimulation during early pregnancy according to BMI levels (**B–D**), including the estimated mean value (lines) and 95% confidence interval (colored regions), descriptive analysis was performed after excluding TPO- Ab positive women.

**Figure 3:**
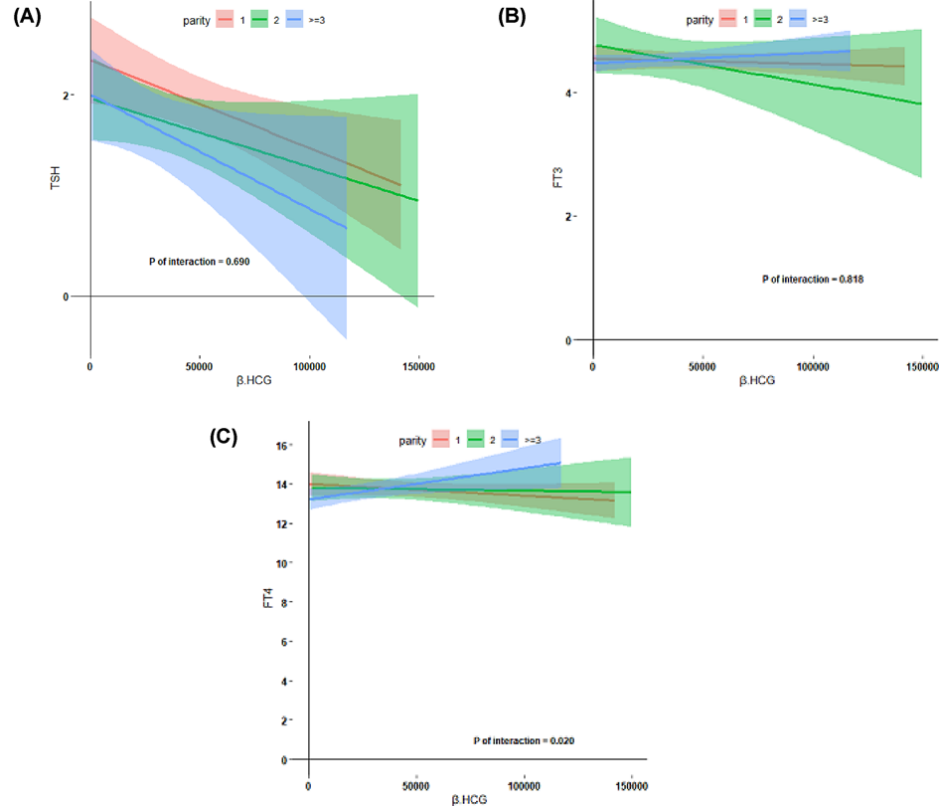
Association between thyroid hormone and parity HCG mediated thyroid stimulation during early pregnancy according to parity (**A–C**), including the estimated mean value (lines) and 95% confidence interval (colored regions), descriptive analysis was performed after excluding TPO- Ab positive women.

**Figure 4:**
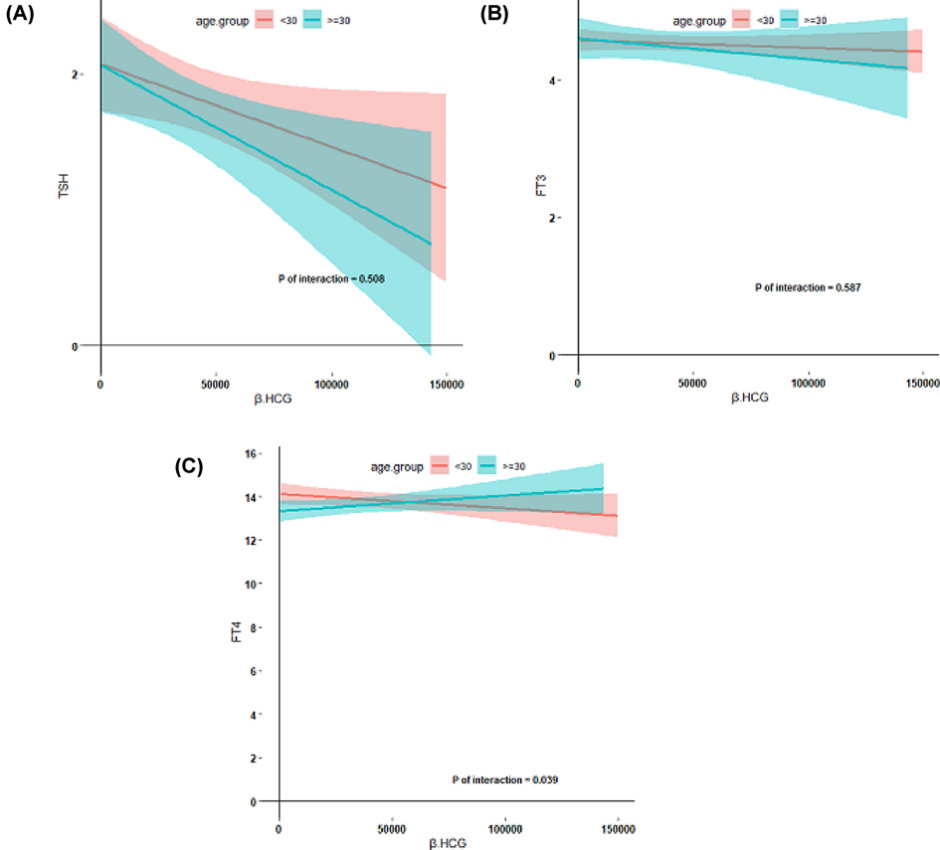
Association between thyroid hormone and maternal age HCG mediated thyroid stimulation during early pregnancy according to maternal age (**A–C**), including the estimated mean value (lines) and 95% confidence interval (colored regions), descriptive analysis was performed after excluding TPO- Ab positive women.

In general, higher BMI was associated with lower FT4 concentrations (Figure 2, *P*=0.005) and there was no association with TSH and FT3 concentrations. In addition, BMI ≤18.5 kg/m^2^ may decrease the response of FT4 to HCG. However, we did not find any difference in TSH and FT3 response to HCG based on BMI. Higher parity was associated with higher FT4 levels (Figure 3, *P*=0.02), which indicates that higher parity is associated with a higher thyroidal response to HCG stimulation. Furthermore, we observed that age >30 years may be a risk factor for a lower thyroidal response to HCG concentrations (Figure 4, *P*=0.04).

**Figure 5:**
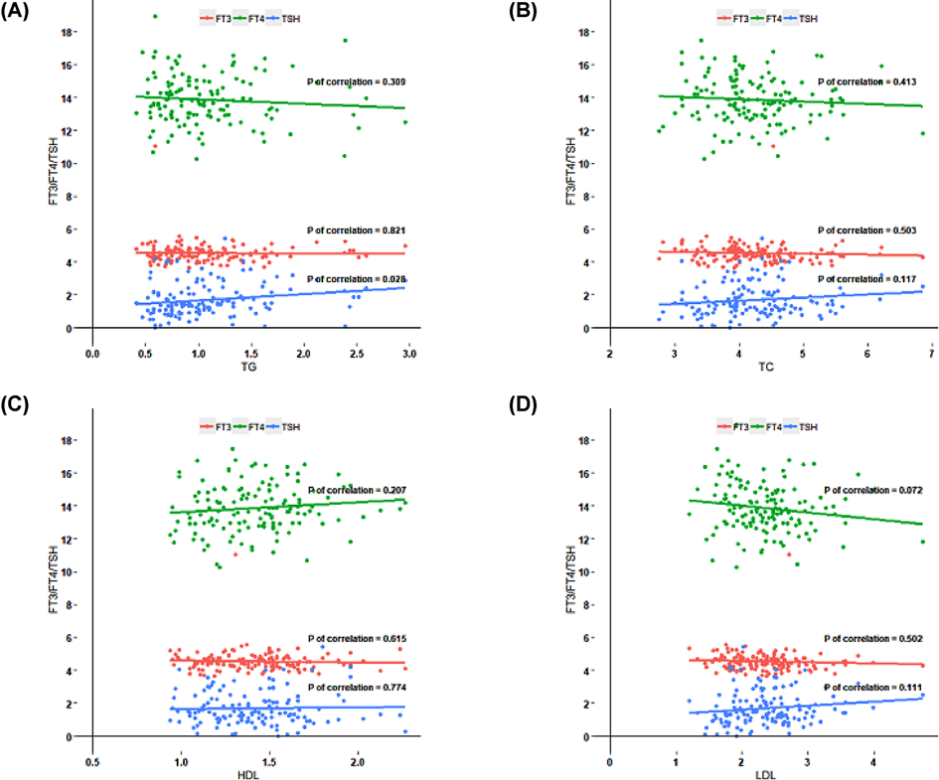
Association between thyroid hormone and serum lipid levels Correlation between different serum lipid levels and TSH, FT3 or FT4 (**A**–**D**).

In multiple regression analysis, TSH was significantly and positively associated with TG (Figure 5, *P*=0.03). However, there was no association between FT4 or FT3 and any of the lipid profiles. After adjustment for age, BMI and diet, there were still no significant associations between FT4, FT3 and dyslipidemia.

## Discussion

The cumulative scientific evidence suggests that gestational thyroid dysfunction can cause adverse pregnancy outcomes, including subclinical hypothyroidism, isolated hypothyroxinemia and TPO-Ab positive [9]. And thyroid hormone replacement therapy can effectively reduce the risk of pregnancy loss among women with subclinical hypothyroidism [20]. Besides, Jansen et al. reported that both high or low maternal free thyroxine concentrations may have adverse effects on infant IQ, grey matter and cortex volume, and overtreatment with LT4 may lead to the potential risk of adverse child neurodevelopment outcomes [10,21]. Therefore, it is important to correctly identify thyroid dysfunction and avoid overtreatment during pregnancy.

Due to the higher affinity of HCG and TSH receptors, the rapid increase in HCG during early pregnancy stimulates the release of serum free thyroxine and a subsequent decrease in TSH concentrations by negative feedback control as compared with the non-pregnant state [21,22]. As gestation increases, HCG levels continue to rise and peak at 10 weeks of gestation, declining slowly thereafter to moderate levels that are maintained until the end of pregnancy. Previous research has shown that median TSH levels at 4-6 weeks of gestation were similar to that of nonpregnant women, but the median concentration of serum TSH decreased significantly after 7 weeks of gestation [23,24]. Similarly, in this Chinese population TSH reference ranges were lower at 7–12 weeks gestation than at 4–6 weeks gestation, and different from reference value recommended by ATA. In addition, thyroid-binding globulin in the blood increases with the change of estrogen levels during pregnancy, which leads to the increase of total TT4 and TT3, but the main risk of adverse pregnancy outcomes and fetal outcomes were caused by free T4, so our study established the reference values of TSH, FT4 and FT3.

Previous studies have observed that with the increase in HCG levels, FT4 showed a positive correlation and TSH showed a negative correlation in a cohort of TPO-Ab negative women, while HCG had no association with FT4 or TSH in a cohort of TPO-Ab positive women [22]. In the current study, we noted some differences in TSH, FT4 and FT3 between the TPO-Ab positive and TPO-Ab negative groups, but they did not achieve statistical significance, potentially due to the small number of women included in the TPO-Ab positive group, differences in ethnicity or iodine status (data not shown). It has been reported that Asian women have higher HCG levels than white or Hispanic women [25], and that thyroid hormone reference ranges vary greatly by ethnicity [26]. One previous study has reported that among Chinese women during the first-trimester of pregnancy the 95% CI for TSH is 0.10 to 4.34 mU/l, and the upper reference limit was only slightly reduced compared with the range reported in the Netherlands [27]. Sample processing and variations in assay methodology may also be important variables in establishing reference ranges. We were not able to measure the isomeride of HCG and therefore β-HCG levels were measured, which may have a different affinity for the TSH receptor and affect the accuracy of the results. In addition, we did not measure the iodine status and urinary iodine concentration, which might also impact thyroid hormone levels.

The 2017 ATA guidelines recommend screening of thyroid function in pregnant women with high-risk factors, which may lead to missed diagnosis if only high risk cases are screened. A multicenter cohort study in China found that screening for thyroid function only in pregnant individuals with associated high-risk factors was likely to lead to missed diagnosis in approximately 81.6% of women with hypothyroidism and 80.4% of women with hyperthyroidism [28]. Nowadays, environmental factors and lifestyle changes have contributed to the increasing incidence of obesity and higher BMI [29], and our study aimed to explore the relationship between BMI and thyroid hormone levels. We observed that higher BMI during early pregnancy was associated with a lower level of FT4, but there was no association with between BMI and TSH or FT3 levels. Besides, BMI ≤18.5 kg/m^2^ may decrease the response of FT4 to HCG. However, we did not find an association between TSH and FT3 response to HCG based on different BMI categories. Irrespective of ethnicity there appears to be a consistent relationship between lower FT4 concentrations and higher BMI during early pregnancy. However, the association between TSH concentration and BMI is less clear [30–32]. Previous studies have observed the interconnection between obesity, thyroid hormone and autoimmunity [33,34]. Higher BMI is associated with higher TSH in non-pregnant women, but the mechanism underlying this association is unclear. Leptin, a hormone-like protein predominantly secreted by adipocytes, is required for the maintenance of TRH expression [35]. Leptin stimulates the expression of the TRH gene through the arcuate neurons projected onto TRH neurons and directly through leptin receptors on TRH neurons [34]. It has been suggested that if leptin secretion is insufficient, the feedback loop between T4 or T3 and the hypothalamus–pituitary–thyroid system will be suppressed. Postmenopausal women with subclinical or overt hypothyroidism (OH) have been demonstrated to have higher levels of leptin [36]. In addition, after adjustment for BMI, supplemental treatment with LT4 can reduce the levels of leptin in OH women, further confirming an association between leptin and thyroid hormone [36]. However, the exact mechanism by which serum leptin affects thyroid hormone remains unclear. Leptin was not measured in the present study, further research is required to determine the relationship between BMI, thyroid function and leptin in early pregnancy.

It has been reported that thyroid hormone have direct effect on hepatic lipid metabolism [37]. Compared with euthyroid, over hypothyroidism has been positively associated with higher TC, LDL and TG, and recovered to baseline values with levothyroxine replacement. Elevated levels of TG have also been shown in clinical hypothyroid and subclinical hypothyroid [38]. In addition, the prevalence of thyroid dysfunction was significantly different in young women with different lipid profiles [39]. The present study focused on the impact of thyroid hormones on lipid profiles in euthyroid adults. It has previously been reported that higher TSH was always accompanied by high levels of TC and TG, even though TSH (5.1–10 mU/l) was only mildly elevated, the levels of TC and LDL were also significantly higher than those with normal thyroid function [40–43]. However, there are few studies about the relationship between thyroid function and lipid disorders in pregnant women. It has been proposed that maternal FT4 was negatively associated with BMI and TG and in patients with TSH ≥2.5 mIU/l an abnormal HDLC/cholesterol ratio was more commonly found [44,45]. In the present study, we found that TSH was positively associated with TG. Thyroid hormone can actively mediate the expression of LDL receptors on the hepatocyte membrane, resulting in increased circulating LDL uptake and reduced circulating cholesterol [37,46]. Hypercholesterolemia in hypothyroidism is mainly due to a reduction in LDL receptor activity, accompanied by the weakened control effect of T3 on sterol regulatory element binding protein 2 (SREBP-2), which modulates cholesterol biosynthesis by regulating the rate-limiting degrading enzyme 3-hydroxy-3-methylglutarylcoenzyme and reductase (HMG-CoA) activity. It was previously proposed that the relationship between TSH and TG was regulated through its regulatory effects on thyroid hormones. In addition, thyroid hormones also increase the activity of lipoprotein lipase, which hydrolyzes TG-rich lipoproteins and accelerates the transfer of cholesterol esters from these lipoproteins to HDL and reduces circulating TG levels. So in hypothyroidism, the increase of TSH may result in the weakening of the above effects, which elevated serum TG. Besides, there are few studies on the effect of thyroid on fetal lipid metabolism. Animal experiments showed that hypothyroidism during pregnancy had no significant effect on insulin resistance and lipid accumulation in the fetus [47]. Further study should focus on the relationship between thyroid dysfunction and maternal lipid metabolism and fetal lipid accumulation.

A major limitation of the study is that the sample size is small and the sample collection was mainly conducted in autumn, which may introduce a selection bias and affect the accuracy of the results. In addition, thyroid hormone and HCG levels were only measured at one time point in early pregnancy, and therefore these reference values can only be used for women ≤12 weeks of gestation, further study is required to establish the reference ranges for the second and third trimesters. Iodine nutritional status and urinary iodine status of participants was not determined, although it may influence thyroid hormone status, but a recent study from China measured serum thyroid function and urinary iodine in an iodine-rich population during early pregnancy and observed that low urinary iodine had no significant effect on mean TSH and FT4 concentrations [48]. A patient conducted questionnaire survey was used to investigate the base information of the participants, such as family history of thyroid disease, history of thyroid disease, drug use, past pregnancy, and as such there may be some information bias. Gestation age was calculated using the last menstrual period, instead of an ultrasound estimate, introducing potential error into the dating.

In conclusion, our study has defined the first trimester-specific reference ranges for serum TSH, FT4, FT3 in a Chinese population, and demonstrated that higher BMI (BMI ≥23 kg/m^2^), high maternal parity (parity ≥3) and increased maternal age (≥30 years) may be risk factors for thyroid dysfunction. Further study should focus on the second and third trimester-specific reference ranges for serum TSH, FT4, FT3 in Chinese pregnant women.

## Data Availability

The datasets generated during and analyzed during the current study are not publicly available but are available from the corresponding author on reasonable request.

## Abbreviations

BMI: body mass index
FT3: free triiodothyroxine
FT4: free thyroxine
HCG: human chorionic gonadotropin
HDL: high density lipoprotein
HMG-CoA: 3-hydroxy-3-methylglutarylcoenzyme and reductase
LDL: low density lipoprotein
SREBP-2: sterol regulatory element binding protein 2
TC: total cholesterol
TG: triglyceride
TG-Ab: thyroglobulin antibody
TPO-Ab: thyroid peroxidase antibody
TSH: thyroid stimulating hormone
